# Novel Noninvasive Spinal Neuromodulation Strategy Facilitates Recovery of Stepping after Motor Complete Paraplegia

**DOI:** 10.3390/jcm11133670

**Published:** 2022-06-25

**Authors:** Ricardo Siu, Edward H. Brown, Samineh Mesbah, Federica Gonnelli, Tanvi Pisolkar, V. Reggie Edgerton, Alexander V. Ovechkin, Yury P. Gerasimenko

**Affiliations:** 1 Kentucky Spinal Cord Injury Research Center, University of Louisville, Louisville, KY 40202, USA; eddie.hb@gmail.com (E.H.B.); mesbahs@ccf.org (S.M.); tanvi.pisolkar@gmail.com (T.P.); alexander.ovechkin@louisville.edu (A.V.O.); yury.gerasimenko@louisville.edu (Y.P.G.); 2 Department of Neurological Surgery, University of Louisville, Louisville, KY 40202, USA; 3 Department of Medicine, University of Udine, 33100 Udine, Italy; gonnelli.federica@spes.uniud.it; 4 School of Sport Sciences, University of Udine, 33100 Udine, Italy; 5 Department of Neurobiology, University of California, Los Angeles, CA 90095, USA; vre@ucla.edu; 6 Department of Physiology, University of Louisville, Louisville, KY 40292, USA; 7 Pavlov Institute of Physiology, Russian Academy of Sciences, 199034 St. Petersburg, Russia

**Keywords:** spinal cord injury, neuromodulation, transcutaneous stimulation, locomotion, rehabilitation, spinal excitability, spinal motor networks

## Abstract

It has been suggested that neuroplasticity-promoting neuromodulation can restore sensory-motor pathways after spinal cord injury (SCI), reactivating the dormant locomotor neuronal circuitry. We introduce a neuro-rehabilitative approach that leverages locomotor training with multi-segmental spinal cord transcutaneous electrical stimulation (scTS). We hypothesized that scTS neuromodulates spinal networks, complementing the neuroplastic effects of locomotor training, result in a functional progression toward recovery of locomotion. We conducted a case-study to test this approach on a 27-year-old male classified as AIS A with chronic SCI. The training regimen included task-driven non-weight-bearing training (1 month) followed by weight-bearing training (2 months). Training was paired with multi-level continuous and phase-dependent scTS targeting function-specific motor pools. Results suggest a convergence of cross-lesional networks, improving kinematics during voluntary non-weight-bearing locomotor-like stepping. After weight-bearing training, coordination during stepping improved, suggesting an important role of afferent feedback in further improvement of voluntary control and reorganization of the sensory-motor brain-spinal connectome.

## 1. Introduction

Trauma to the spinal cord usually leads to damage of ascending and descending spinal tracts, interrupting the flow of information to and from the brain. This can lead to partial paralysis if some tracts are preserved and complete paralysis if none or a small percentage are preserved. A large body of work has shown partial recovery of locomotion after SCI through multiple approaches. Locomotor training has been used widely in rehabilitation and has been examined in animal studies [1,2] and human subjects with incomplete paraplegia [3,4,5,6]. Pharmaceutical agents have also demonstrated successful restoration of partial function in animal studies [7,8] and in human subjects with motor complete paraplegia [9]. More recently, electrical stimulation of the spinal cord through invasive epidural electrical stimulation in animals [10,11,12,13] and humans [14,15,16,17] and non-invasive transcutaneous electrical stimulation in humans with incomplete and complete paraplegia [9,18,19,20], have shown very promising results. Finally, electrical stimulation of peripheral nerves and muscles in humans with incomplete paraplegia [21,22,23] have also shown evidence of functional recovery. This is likely through the neuromodulatory effects that these approaches bring about and the subsequent effect of enhancing neuroplasticity, reawakening dormant networks and/or creating new functional connections within and between spinal networks [24,25,26]. By merging multiple approaches to engage activity-dependent plasticity mechanisms, synergistic effects are likely to be generated, thus facilitating recruitment and coordination of motor pools. If performed appropriately, this may lead toward recovery of locomotor function after severe spinal cord injury.

Here, we explored the use of a combinatorial approach of non-invasive multi-site spinal cord transcutaneous electrical stimulation (scTS) with activity-based non-weight-bearing training (nWBT) and weight-bearing training (WBT) to enhance neuroplasticity of the spinal pathways and networks to facilitate stepping behaviors on a 27-year-old participant with a spinal cord injury (SCI) at T8-T11 classified as American Spinal Injury Association Impairment Scale (AIS) level A. This case study encompasses the first phase of a broader multi-phase study. In-silico simulations in a virtual phantom were performed to assess the specific neural structures targeted by each individual stimulation site. Functional assessments were carried out to evaluate his ability to voluntarily move his legs without weight-bearing in a Gravity Neutral Device (GND) and during weight-bearing on a Body-Weight Support Treadmill (BWS-TM) to assess activation of his leg muscles through electromyography (EMG) and kinematics. Neurophysiological assessments evaluated the state of supra- and sub-lesional connectivity through multiple pathways and systems by using a paradigm of supra-lesional conditioning of motor evoked potentials (MEPs). MEPs were triggered through single-pulse transcutaneous electrical spinal stimulation at the lumbar spinal cord after a conditioning stimulus was delivered above the site of injury [27]. Overall, these assessments showed that locomotor function improved after 3 months of locomotor training with scTS, likely through changes in the spinal pathways and networks. These results suggest that rehabilitative strategies, when combined with scTS, can facilitate recovery of function after SCI.

## 2. Materials and Methods

**Figure 1:**
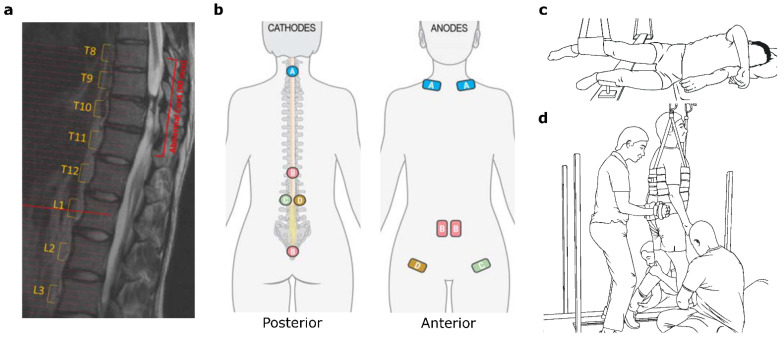
Participant information and experimental setup. (**a**) MRI showing region of the spinal injury. The injury spans the T8—T11 (vert) levels. (**b**) Electrode placement for scTS. Cathode/Anode pairs are color coordinated and labeled according to their respective pair. Cervical scTS at C3/C4 is paired with bilateral clavicle anodes (A); continuous scTS at T11/T12 and Co1 are paired with abdominal anodes (B), and left and right L2/L3 are paired with ipsilateral anodes at the anterior superior iliac spine (C, D respectively). (**c**) Participant positioning in the Gravity Neutral Device for nWBT. Both legs are suspended to minimize the effect of limb weight on limb movement. (**d**) Participant in the body weight-support treadmill with manual facilitation of hip and leg movement by locomotor trainers for WBT. Diagram shows trainer hand positioning to facilitate hip stability, knee movement, and foot positioning during locomotor training.

The participant is a 27-year-old Caucasian male that presented with a motor complete paraplegia after an incomplete SCI at T8-T11. Extent of the injury was verified through magnetic resonance imaging (Figure 1a). The participant was classified as AIS-A with a neurological level of injury at T2 with a partial motor and sensorial preservation zone up to T7 and T9, respectively, as determined by an International Standards for Neurological Classification of SCI (ISNCSCI) examination. Damage to the spinal cord was due to indirect trauma lateral to the spinal cord 5 years prior to study enrollment. No supportive vertebral hardware was implanted. The participant reported the regular use of cannabis for pain control and recreational purposes. There was no reported intake of other pharmacological forms of pain control. The participant did participate in a study assessing the acute effects of 5-HTP during the first week of training. However, there is no evidence to support that this had a significant effect after 1 or 3 months of training. Additionally, kinematic data was collected in a non-weight bearing setting on a height-matched non-disabled participant for reference (Supplementary Figure S1). All of the human assessments and interventions were approved and carried out in accordance with the University of Louisville Institutional Review Board. Informed consent was obtained from the participants to carry out this study.

### 2.1. Spinal Cord Transcutaneous Electrical Stimulation

Previous studies have shown that standing and step-like movement patterns of lower limbs can be elicited by electrical stimulation at the L1/L2 and T11/T12 vertebral levels, respectively [18,28,29,30]. However, early mapping sessions during non-weight bearing in the GND showed that a more caudal site, L2/L3, with anodes placed along the inguinal ligament provided a more consistent and effective knee extension than the L1/L2 site suggested by previous studies (Supplementary Figure S2). Additionally, to provide function-specific stimulation, L2/L3 electrodes were placed bilaterally for left-right specificity. Stimulation at these sites was delivered in an alternative fashion with a fixed pulse-train duration of 2 s and a cycle period of 6 s or sub-motor threshold, depending on the training task, in nWBT, and in a closed-loop manner in WBT where gyroscopes attached above the knee triggered stimulation during the stance phases of the gait cycle for each leg. Other studies have also shown that scTS of the cervical area can lead to the facilitation of voluntary behaviors [28,31]. Lastly, stimulation of the sacral afferents has been shown to be an effective neuromodulator of the lower limb muscles [32,33]. We attempted to replicate this effect through scTS at the sacral-coccygeal level.

Thus, to comprehensively modulate the entire locomotor network within the spinal cord, scTS was delivered at five different locations, midline C3/C4, midline T11/T12, bilaterally at L2/L3, and midline on the sacral-coccygeal region. Circular cathodes (3.175 cm dia PALS^®^, Axelgaard, Fallbrook, CA, USA) were placed in the aforementioned locations, while anodes (5.08 cm × 8.89 cm PALS^®^, Axelgaard, Fallbrook, CA, USA) were placed on the clavicles for the cervical cathodes, on the rectus abdominis for the T11/T12 and sacral-coccygeal cathodes, and ipsilaterally along the inguinal ligament for the respective L2/L3 cathodes. Placement of the cathodes and anodes can be seen in Figure 1b. A BioStim-5 Stimulator (Cosyma Inc., Denver, CO, USA) delivered scTS consisting of biphasic pulses at the C3/C4 and T11/T12 locations, and of monophasic pulses at the other locations. Monophasic pulses evoked discomfort but evoked better functional outcomes, hence biphasic pulses were delivered at the sites above the level of sensory perception to prevent discomfort. Pulse width duration was 1 ms for all sites and stimulation frequency was set to 30 Hz for all midline sites and at 40 Hz for the L1/L2 sites. These parameters were determined through multiple mapping sessions prior to training. A modulating carrier frequency at 5 kHz was used to minimize sensory sensitivity to scTS [34]. The current amplitude was determined at the start of each session and set to be at motor threshold unless the participant reported discomfort from the stimulation. The motor threshold was determined by EMG, visual observation of muscle contraction and/or movement.

### 2.2. Locomotor Training

The participant underwent locomotor training with scTS five times a week for three months. Each training session lasted approximately 1 h. Locomotor training was divided into two phases: an initial phase of one month in which the participant received scTS with nWBT in a GND [28] carrying out voluntary tasks and passive locomotion through trainer assistance, and a second phase lasting two months in which scTS was delivered during WBT on a BWS-TM [35].

When in the GND (Figure 1c), the participant was lying recumbent with their legs suspended to reduce the effort required to move the lower limbs against gravity. Here, the participant was asked to perform two tasks: to attempt voluntary hip and knee extension for each leg and to swing their legs in a rhythmic step-like manner (air-stepping), adding arm swings during their attempt. Hip and knee extension tasks consisted of four sets of three to four repetitions per leg, with rest between sets. The air-stepping task consisted of two sets, each lasting four to five minutes, depending on participant endurance. Passive conditioning with trainer assistance for three minutes was delivered between air-stepping sets. Stimulation was delivered concurrently and remained the same between tasks with the exception of the L2/L3 bilateral sites, where scTS was delivered alternatively as described in the previous section during leg extension tasks but was set to continuous stimulation at a sub-motor threshold level during air-stepping due to lack of a reliable triggering mechanism. 

During WBT in the BWS-TM (Figure 1d), the participant was asked to attempt stepping while trainers are positioning and assisting hip, leg, and ankles. These sessions are further subdivided into 3 bouts at a fast treadmill speed (1.5–2.0 m/s) to elevate the overall excitability of the locomotor network and thus train the automaticity of the locomotor spinal central pattern generator (CPG), and 2 to 3 bouts at slow speeds (0.5 m/s) to drive voluntary effort and thus strengthen corticospinal pathways. Rest periods of one to two minutes were allowed between the bouts. Body weight support was provided initially at 40% body weight and could be reduced to 35% at the end of the training period based on locomotor trainer feedback. During WBT, scTS was delivered as described in the previous section with gyroscope-triggered stimulation at the L2/L3 bilateral sites.

### 2.3. Kinematic Data and EMG Data Collection and Analysis

To assess kinematic behavior in a non-weight bearing setting, the participant lies recumbent on their left side and reflective markers are placed at the right shoulder, right hip, right knee, right ankle and right toe to obtain hip, knee, and ankle angles of the right leg. The participant was asked to attempt to move his legs in a step-like manner, focusing on evoking and maintaining this behavior. Kinematic data was collected at a sampling rate of 100 Hz and analyzed using a 5-camera motion capture system with Cortex kinematic analysis software (Motion Analysis Co., Rohnert Park, CA, USA). Movements were extracted from data collected 10 s after the start of each air-stepping task. Each extracted period lasted for 10 s. The Cortex kinematic analysis software superimposes the spatial data from the reflective markers on a skeletal frame and utilizes built-in functions to output specific joint angles. Kinematic data without and with scTS, using stimulation parameters as described above, was collected at baseline, after GND training, and after BWS-TM training.

Electromyography (EMG) of the lower limbs was collected in the BWS-TM while performing weight-bearing stepping to assess changes in muscle activation after BWS-TM training. EMG of the rectus femoris (RF), vastus lateralis (VL), medial hamstring (MH), tibialis anterior (TA), medial gastrocnemius (MG), and soleus (SOL) muscles was collected using surface EMG electrodes (MA400, Motion Labs Systems, Baton Rouge, LA, USA) at a sampling rate of 2000 Hz. EMG electrodes were placed above the largest cross-section of the muscle (“muscle belly”) and oriented following the direction of the muscle fibers [36]. EMG was filtered using 4th order Butterworth bandpass filter with a passband of 30 Hz to 450 Hz and an adaptive filter to remove background noise and stimulation artifact, when present [37]. The adaptive filter removes frequency components found in a baseline template, in which stimulation artifacts and background noise are present but muscle activity is absent, from the overall EMG signal, allowing for specific removal of frequencies only found in the baseline template. EMG was analyzed to obtain total power and median frequency before and after WBT [38].

Muscle coordination between agonist and antagonist pairs was assessed by calculating the Pearson’s correlation coefficient (PCC) during peak activation of the normalized EMG envelope between muscle pairs [39,40]. EMG was normalized to the average peak activation across cycles since a maximum voluntary contraction is not possible in the SCI population. The normalized EMG was then smoothed using spline interpolation across local maxima separated by at least 100 ms. The long duration of the window was used to attenuate motor unit spikes prevalent in the EMG signal. Simultaneous peak activation in muscle pairs lead to a high PCC; low PCC suggests low coupling between both muscles, and a high negative correlation suggests that there is coupling albeit with a 90° phase difference, suggesting antagonistic behavior. To assess overall positive changes in coordination after WBT, a relative PCC value was obtained by inverting the PCC of antagonist muscle pairs, such that a value of 1 dictates appropriate antagonistic activation. A positive change in the relative PCC dictated improvement in muscle coordination while a negative change indicated decline. See Supplementary Figure S3 for further details.

### 2.4. Supraspinal Conditioning of Motor Evoked Potentials

Modulation of MEPs at the lumbar region through conditioning stimuli has been used in several studies to evaluate the patency of descending tracts through a damaged spinal cord [17,27,41]. Here, we utilized these approaches to assess MEP conditioning through cervico-lumbar pathways (CLPs), the propriospinal system (PSS), and reticulospinal pathways (RSP). Additionally, a novel paradigm to evaluate the state of the corticospinal pathways (CSP) using a task-driven paradigm was also developed.

A conditioning stimulus was delivered at a site above injury that pertains to each pathway while the participant is laying supine. To condition through the PSS, a stimulus was delivered at the ulnar nerve of the right arm. For the CLPs, a single pulse delivered through a scTS electrode located midline at the C3/C4 vertebral level served as the conditioning stimulus. To condition through the RSP, an acoustic startle reflex was elicited using a 1000 Hz tone at 80 dB [42]. To condition the locomotor network through CSP, the participant was asked to perform six different motor tasks at the onset of an auditory cue for a duration of 3 s (Supplementary Figure S5). The tasks were left plantarflexion, right plantarflexion, left dorsiflexion, right dorsiflexion, bilateral knee extension, and bilateral knee flexion. Each task was performed three times.

To elicit an MEP in each of the conditioning modalities, a secondary stimulus was delivered at a site below the injury at L1/L2 vertebral level 90 ms after the stimuli for the CLPs, PSS, and RSP [41]. For CSP conditioning, a stimulus was delivered at a site below the injury at L1/L2 during the voluntary task (1000 ms after tone onset). The amplitude of the second stimuli was selected such that activation of all of the muscles was observed but was not at maximum or minimum of any muscle to ensure that both inhibition and facilitation of MEPs can occur. 

A minimum of three conditioned responses, along with control MEPs, were collected at each time point, before nWBT, after nWBT, and after WBT for CLP, PSS, and RSP conditioning and after nWBT and after WBT for CSP conditioning. A difference in the area under the curve (AUC) of the MEP with respect to the control stimulus indicated modulation through each respective system or pathway. Muscle responses were collected through Spike2 software (CED Ltd., Cambridge, England) at a sampling rate of 5000 Hz. MEPs were collected from the RF, VL, MH, TA, MG, and SOL muscles. The data was processed and analyzed through MATLAB 2020A (Mathworks Inc., Natick, MA, USA). Assessment of the CSP was performed only after nWBT and after WBT. Given the limited data set, no statistical analysis was performed.

### 2.5. Computational Modeling

Current field distribution simulations were performed to elucidate the neural structures targeted by scTS at each of the stimulation sites. Ohmic quasi-static simulations were performed using a finite element model approach (Sim4Life, Zurich Med Tech, Zurich, Switzerland) in a virtual model (Yoon Sun ViP 4.0, IT’IS Foundation) with tissue-specific electrical properties [43]. Current amplitudes used in the simulation matched average amplitudes used experimentally. The current field density was used to assess the foci of stimulation with simultaneous scTS.

### 2.6. Statistical Analysis

Given the nature of this case study, statistical analysis is limited. However, statistical significance of differences in EMG total power and median frequency before and after WBT were calculated. A non-parametric Wilcoxon signed-rank test was used after checking for normality. The area under the curve for the MEPs in the neurophysiological studies was also statistically analyzed using a Wilcoxon signed-rank test.

## 3. Results

### 3.1. Computational Modeling of Current Density Induced by Multi-Site scTS

**Figure 2:**
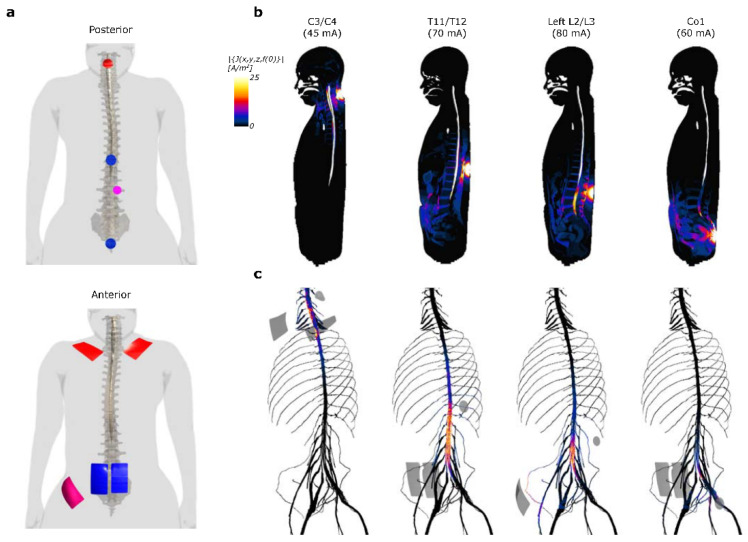
Computational simulations in a virtual phantom show differential distribution of charge density after scTS at different stimulation sites. (**a**) Placement of the cathodes and their respective anodes on the virtual phantom. (**b**) Sagittal view of the current density for each stimulation site show segment-specific stimulation. (**c**) Current density at the surface of the spinal cord, roots, spinal nerves, femoral, and sciatic nerves show the neural structures targeted by each stimulating site. Anodes/cathodes for each stimulation site are shown in grey.

Computational modeling of the scTS electric current distribution throughout the virtual phantom shows that, while relatively widespread, scTS at the different sites (Figure 2a) is able to stimulate distinct neural structures (Figure 2b,c). The C3/C4 cathode increases the current density around the C5 spinal segment but is distinctively concentrated along the C5 and C6 dorsal roots. Stimulation between the T11/T12 vertebrae causes a spike in concentration along the spinal cord L2 segment, but also likely stimulates all of the dorsal and ventral nerve roots below the spinal cord L2 segment. Similarly, the L2/L3 lateral cathode stimulates a vast majority of the lumbar nerve roots, however, only below the L4 level and with additional lateral activation of the left femoral nerve and L1 spinal cord nerve. Finally, stimulation at the Co1 predominantly leads to activation of the S3–S4 roots.

### 3.2. Improvement in Hip and Knee Kinematics during Non-Weight Bearing Stepping

**Figure 3:**
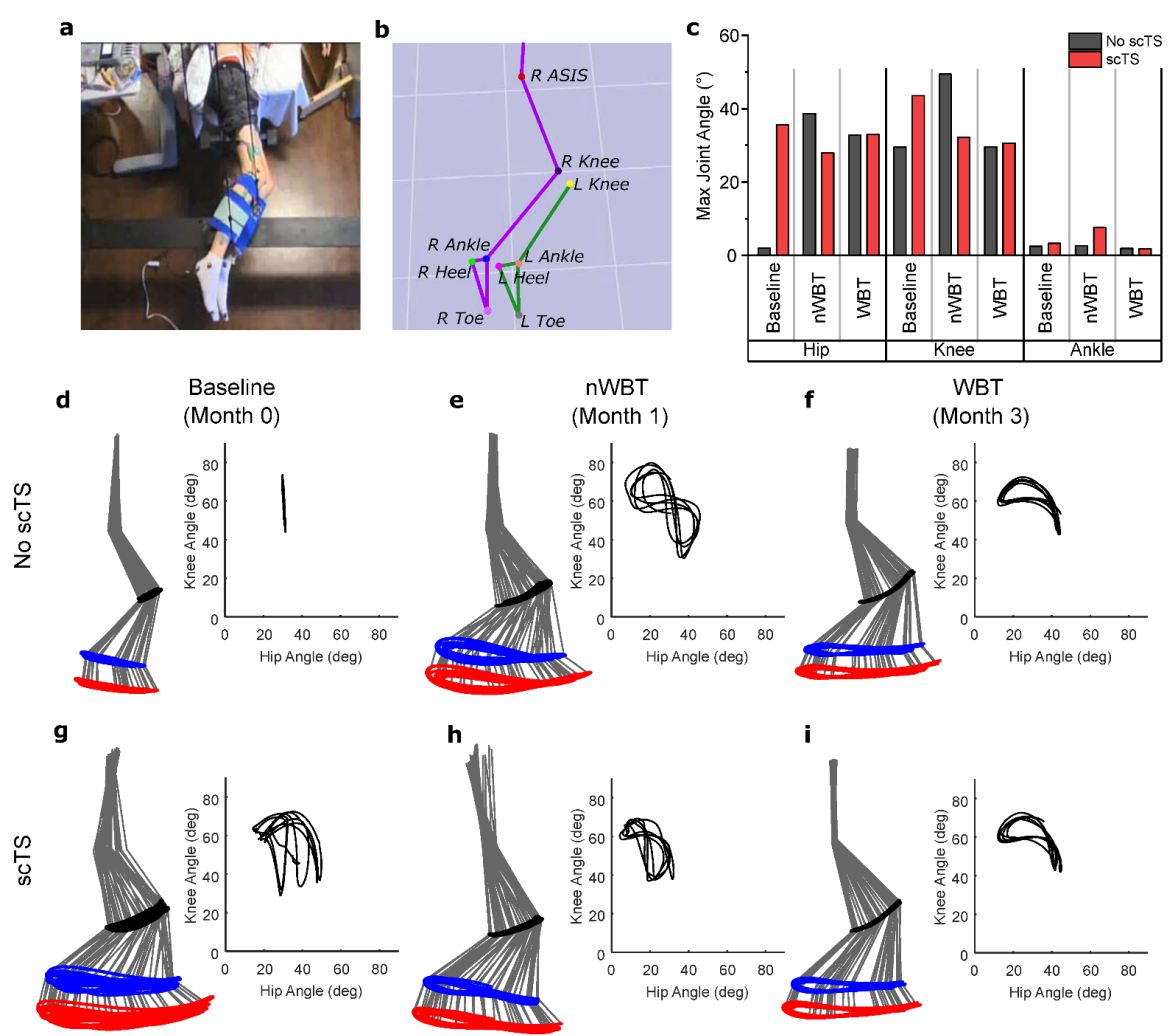
Kinematics of the right leg during a voluntary step-like locomotive behavior indicate improved hip-knee coordination after training, with differences between non-weight bearing training and weight-bearing training. (**a**) The participant was positioned in the gravity neutral device with reflective markers placed at major bony landmarks. (**b**) 3-D motion analysis software converted marker data to hip and knee joint angles to assess locomotor behavior. (**c**) Maximum joint angles without and with scTS and at baseline, post nWBT and post WBT. Stick diagrams showing kinematics of the hip, knee, and ankle joints and cyclograms showing hip-knee joint coordination at the baseline (**d**,**g**), post nWBT (**e**,**h**), and post WBT (**f**,**i**) timepoints indicate improvements in range of motion and in limb control without and with scTS.

Kinematic data collected in a non-weight bearing setting (Figure 3a,b) showed distinct changes in maximum joint angles (Figure 3c) and hip-knee coordination both immediately after scTS, due to locomotor training. Baseline data showed limited joint movement, with only a 1.9° range in hip angle and a 29.5° range in knee angle (Figure 3d). After nWBT (Figure 3e), however, these angles ranges increased to 38.7° and 49.4°, respectively. After WBT (Figure 3f), there was a reduction in the total hip angle to 32.9° and 29.6° at the knee, however, the kinematic pattern follows a more coordinated stepping pattern that more closely resembles the ellipsoid knee-hip dynamics observed in a non- injured volunteer in the GND (Supplementary Figure S1). The angles of the ankle joint showed no considerable change and were less than 5° at all time points. With the addition of scTS during baseline (Figure 3g), hip angles increased dramatically, however, coordination between the hip and knee was not optimal and was highly variable. After nWBT (Figure 3h), joint angles decreased when scTS was applied, however, there is an improvement in the overall hip-knee coordination as the “Figure 8” pattern observed without scTS transitions towards a more normal ellipsoid pattern. Finally, after WBT (Figure 3i), there was no evident change in joint angles and the appropriate pattern observed without scTS remained.

### 3.3. Increase in EMG Power and Muscle Coordination during Weight-Bearing Stepping

**Figure 4:**
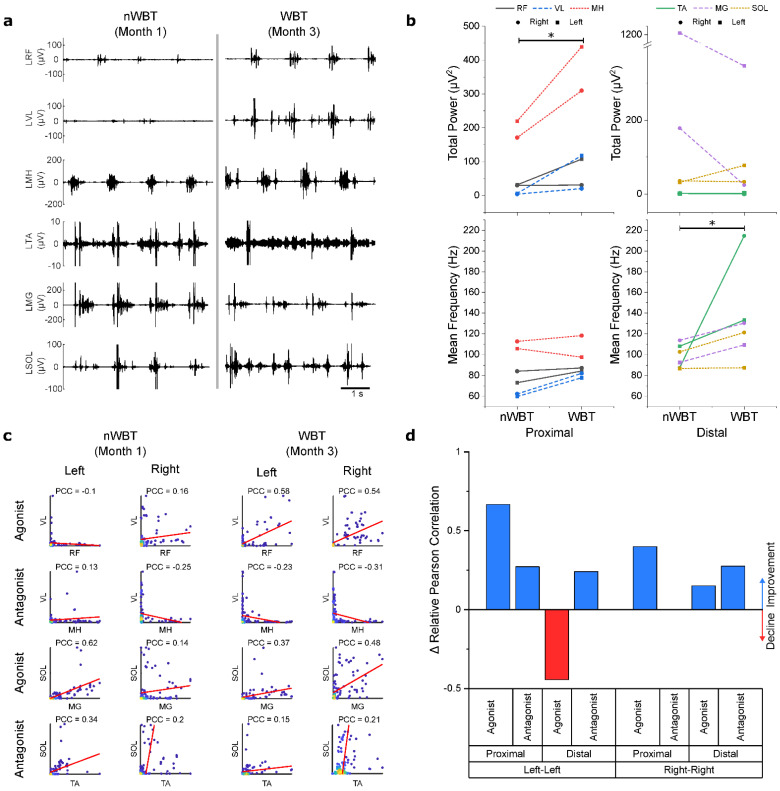
Electromyography collected during stepping assessments in Body Weight Supported Treadmill before and after weight-bearing training without stimulation indicate improvements in muscle activation and coordination. (**a**) EMG traces of the left leg show improvement in quality of EMG bursts in the proximal muscles. (**b**) Overall total power and mean frequency of the grouped proximal and distal muscles show statistically significant improvements (indicated by *) in total power of the proximal muscles and mean frequency in the distal muscles after WBT but do not show significant difference in total power of distal muscles nor in mean frequency of proximal muscles. (**c**) Correlation between normalized peak EMG of functional muscle pairs show changes in coordination between agonist and antagonist muscle pairs in both left and right legs before and after WBT. The Pearson’s Correlation Coefficient (PCC) is shown for each muscle pair. (**d**) Changes in Relative PCC between muscles sets after WBT show a general improvement across muscle pairs except for the left distal agonist pair. A positive change indicates improvement in muscle coordination, while a negative change indicates decline in coordination.

Assessments on the BWS-TM showed changes in EMG power and mean frequency, as well as changes in muscle activation patterns between muscle sets. Figure 4a shows EMG traces of the left leg muscles before and after WBT training. Analysis of EMG properties (Figure 4b) shows that WBT led to an increase in total power of EMG signals recorded from the proximal muscles (*p* = 0.031). The distal muscles, however, did not show a significant difference in power (*p* = 0.313). There was no significant difference in mean frequency for the proximal muscles (*p* = 0.156), but distal muscles did show a significant increase (*p* = 0.031).

The correlation between agonist and antagonist muscle pairs after nWBT and WBT shows that there is an overall increase in coordination (Figure 4c), with the relative correlation increasing among all of the muscle pairs except for the distal agonists (Figure 4d). When assessing coordination between muscle pairs, an average increase of 0.53 was observed in the relative Pearson’s correlation coefficient difference for bilateral agonist proximal muscles, and an average increase of 0.14 observed in antagonistic muscles. Distal muscles saw a decrease of 0.15 in agonist muscles, but an increase of 0.26 compared to before WBT. A more detailed matrix of relative PCC changes can be found in Supplementary Figure S4.

### 3.4. Facilitation of Supraspinal Conditioning of Motor Evoked Potentials

**Figure 5:**
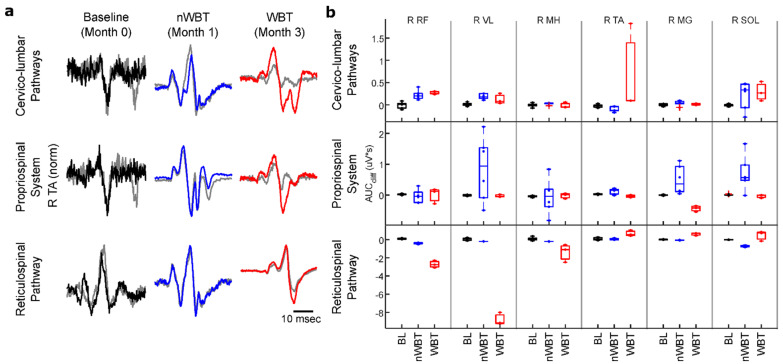
Motor evoked potentials after supra-spinal conditioning through non-specific cervico-lumbar pathways, propriospinal system, and reticulospinal pathways do not show modulation before training (baseline, black), but do show modulation after non-weight bearing training (nWBT, blue) and weight-bearing training (WBT, red). (**a**) Motor responses without conditioning (grey) and with supra-lesional conditioning across timepoints show changes in modulation of the right tibialis anterior (R TA) muscle after training. (**b**) Training has a differential effect on the difference in the area under the curve of the motor evoked potentials after modulation when compared to control responses. This differential effect is also observed across multiple muscles. Boxplots show median values and lower and upper quartiles.

Neurophysiological studies assessing the conditioning of sub-lesional locomotor MEPs suggest that training with scTS, without or with weight-bearing, can lead to re-emergence of supra-lesional modulation of the locomotor network differentially through the multiple spinal pathways. Figure 5a shows average non-conditioned and conditioned responses of the right tibialis anterior (TA) muscle for each conditioning paradigm.

Figure 5b shows that the percent change in area under the curve (AUC) relative to non-conditioned MEPs differentially changed between muscles, across different pathways tested, and after nWBT and WBT. The largest overall change in MEP responses were observed when conditioning MEPs through the reticulospinal pathways (RSP). Albeit, contrary to what we expected, the changes in MEP conditioning through the RSP are mostly of an inhibitory nature. However, since the non-specific cervico-lumbar pathway (CLP) modulation did cause a largely facilitatory change, it is possible that the inhibitory response observed in RSP modulation is due to changes at the supraspinal level rather than the result of spinal neuroplasticity. Changes in modulation through the propriospinal system (PSS) showed increased modulation after nWBT but returned to baseline values in most muscles after WBT.

**Figure 6:**
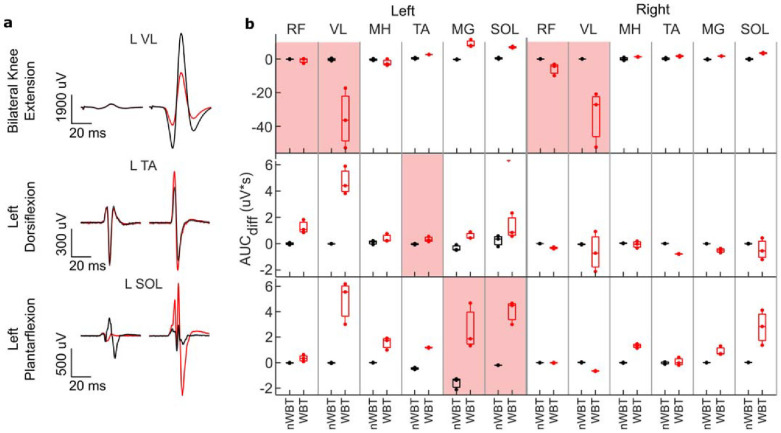
Voluntary modulation of motor evoked potentials during bilateral knee extension and unilateral plantarflexion and dorsiflexion of the left ankle show task−specific modulation after treadmill training. The contralateral leg was at rest during unilateral tasks. (**a**) Spinal cord evoked motor potentials of one of the main muscles involved in each voluntary task show a change in responses after weight−bearing training. (**b**) Weight-bearing training led to a change in the difference of the area under the curve compared to control pulses across all muscles for each voluntary task. Different tasks also modulated the motor responses differentially. The shaded area indicates the main muscle active during these tasks.

Corticospinal conditioning through the voluntary intent tasks showed that after nWBT, modulation of MEPs was minimal or not present in the main muscles involved during these tasks. After WBT, there is evidence of modulation of the MEPs in all main muscles (Figure 6a). Paradoxically, while still showing modulation, the left vastus lateralis (VL) muscle shows inhibition rather than facilitation of the MEPs during knee extension. Figure 6b shows the AUC when compared to control pulses at each timepoint across all muscles. After WBT, most muscles show a change in responses across behaviors.

Bilateral knee extension shows a significant inhibition of the VL muscle motor response, while a left plantarflexion task increases the response of left plantarflexor muscles more so than the right plantarflexor muscles. Left dorsiflexion also shows facilitation of the left VL, medial gastrocnemius (MG), and soleus (SOL) muscles, but inhibition of the right VL muscle. Supplementary Figure S6 shows similar results across bilateral knee flexion and extension and unilateral ankle dorsiflexion and plantarflexion for both left and right ankles.

## 4. Discussion

A novel strategy of non-invasive multi-segmental spinal cord transcutaneous electrical stimulation was developed to provide multi-functional enabling of the locomotor-related neuronal networks for facilitation of stepping recovery after motor complete paraplegia. Transcutaneous electrical stimulation of the spinal cord has shown in multiple studies to neuromodulate posture and locomotion [18,20,28,29,30]. Furthermore, animal studies have shown that spinal stimulation can also evoke plasticity at the spinal cord level, improving connections and reactivating dormant networks [44]. Use-dependent training has also been shown to be an effective method for increasing spinal plasticity after SCI [4,35]. The strategy developed here leverages these findings to restore locomotor function through spinal stimulation and use-dependent training. The results of the present case study show a general increase in locomotor function through improved muscle activation and coordination, while also showing increased modulation by supra-lesional pathways, highlighting the effectiveness in this strategy to modulate spinal networks and restore bidirectional spinal-supraspinal connectivity. This study confirmed that locomotor behavior and response after training were dependent on sites of stimulation and presence of proprioceptive feedback: weight bearing.

This multi-site stimulation strategy targeted multiple functional spinal areas to assist in the recovery of locomotor function. It has been suggested that electrical stimulation above the site of injury at the cervical level can potentially regulate the brain-spinal connectome and reactivate descending dormant systems, that electrical stimulation at T11/T12 activates the locomotor network to induce locomotion, and that sacral-coccygeal (Co1) stimulation has been used to modulate the locomotor network [9]. Finally, electrical stimulation at L2/L3 has been applied to regulate the postural functions and integrate postural-locomotor coupling. When merged, these inputs can synergistically regulate the locomotor network to facilitate recovery of stepping through a multi-system integrative approach. 

Computational simulations showed that the multi-site scTS configuration used in this study targets multiple sites along the spinal cord and various spinal roots (Figure 2). Modeling scTS at the C3/C4 and T11/T12 levels showed a clear concentration of current along the surface of the spinal cord, however, sites L2/L3 and Co1, showed high current density concentrations along the cauda equina, with Co1 stimulating predominantly a subset of these spinal roots (S2–S4). While these results show that the T11/T12 and the lateral L2/L3 seem to target very similar structures with significant overlap, data collected during nWBT training showed very distinct behaviors when stimulating independently. Stimulation at the lateral L2/L3 sites showed noticeable facilitation of unilateral knee extension. This was not observed with stimulation at T11/T12. Although the current computational model does not differentiate specific spinal pathways nor inter-neuronal networks, it serves to link the stimulation sites to a motor response to different weight bearing conditions as well as to unique sources and levels of proprioception.

The application of stimulation during non-weight bearing stepping led to a different kinematic effect across different time points (Figure 3c–i). It is possible that scTS played different roles across time points as a result of neuroplastic changes. Prior to training, it increased the overall locomotor muscle activity. After nWBT, scTS may have shifted to improving control of the hip joint, reducing angles as a result. After WBT, since afferent information during weight bearing has shown to play a crucial role in locomotion [45,46], scTS may instead serve as a mechanism that provides a more effective balance in the levels of inhibition in contrast to excitation, resulting in improved reciprocal inhibition and coordination. Subjects receiving spinal neuromodulation of emphasize that it facilitates stepping [47].

Training with scTS showed clear improvements in hip and knee function. Kinematics showed that after nWBT, hip angles dramatically increased during voluntary stepping in the GND. However, the hip-knee cyclograms show a “Figure 8” pattern (Figure 3e) that suggests that control of the knee joint is dependent on hip mechanics more so than through voluntary control of the joint. This is further reiterated through EMG recordings performed during stepping in a BWS-TM, where knee flexor activity overshadows that of knee extensors (Figure 4a). Furthermore, EMG coordination analysis also showed a low correlation between knee extensors (Figure 4c), suggesting impaired proximal agonist muscle coordination, which may prevent adequate knee extension. After BWS-TM training, this pattern disappears and a pattern showing a more active knee movement appears (Figure 3f). This implies more involvement of the knee joint which is now being actively controlled to resist the force of hip momentum. This final hip-knee angle pattern closely resembles a traditional hip-knee cyclogram seen in non-disabled individuals (Figure 3c) compared to the pattern at previous timepoints [45,46].

EMG after BWS-TM Training collected in the BWS-TM confirms a comparatively more active knee joint muscles by showing a substantial increase in VL EMG power and improved correlation between right VL-RF activation (Figure 4). MEPs collected during voluntary tasks also showed clear VL modulation when compared to control MEPs. However, while this shows that supra-lesional connections to the locomotor network are present, inhibition is observed rather than facilitation; a possible reason being changes in afferent input during these voluntary tasks when compared to what the setting is during BWS-TM Training, where weight-bearing leads to loading of the muscles. This difference in afferent input may be the cause of this inhibitory response, rather than excitatory response. It is also possible that the cortex has yet to completely associate a desired voluntary task to specific behaviors and thus selective activation of muscles is still not optimal. Further ongoing training could elucidate this contraindicative behavior.

The improvement in hip and knee kinematics and proximal muscle activity after WBT was substantial, particularly having begun with complete paralysis. Improvement in ankle movement, however, was minimal. No change was observed in the ankle angles during GND assessments but an increase in SOL power was observed and a large decrease in TA activation occurred. We surmise that this is due to the functional aspect of WBT, where focus is placed on training for standing and for support during stance. During these activities, plantarflexion is desired, maintaining SOL and MG activity constant to prevent forward bend of the ankle. Across 40 training sessions, there was an overall decrease in dorsiflexor muscle activity and an increase in plantarflexor activity. This can be further shown when assessing facilitation of MEPs, where, during dorsiflexion, we observed much larger facilitation of MG and SOL and relatively smaller facilitation of TA.

The supraspinal conditioning of MEPs below the injury indicates the restoration of cross-lesional spinal pathways as this is consistent with supraspinal network influence, although the conditioning effect could also reflect elevated levels of excitability of spinal networks as well. Changes were observed across all of the tested pathways, however, the RSP proved to have undergone the most change, while the PSS showed minimal changes (Figure 5). Of note were the changes in the CSP, where each task led to differential modulation across muscles, which was not evidently observed prior to WBT (Figure 6). This task-specific modulation of MEPs is evidence that new pathways coming from the cortex have been created or that dormant pathways across the lesion have been reactivated. This paradigm could also be used in future studies to assess the specificity by which cortical drive can facilitate specific muscle groups below the level of the injury, with specificity increasing as locomotor function is restored.

This work, being a case study, has inherent limitations such as having a single sample that belongs to the already highly variable SCI population. As the work reported here is part of a larger study, future results will provide a better assessment of the effectiveness of scTS with locomotor training once more participants have undergone the training. Also, due to limitation of the study protocol at the initial point of the study, there is a lack of baseline data for the EMG assessments in the BWS-TM and of the CSP conditioning assessment. However, kinematic data during GND assessments at baseline show very minimal activity, allowing us to assume that there would be very low or no activity and modulation of the leg muscles. This allows us to make the comparison only between the post-GND time point and post-BWS-TM time point. Another limiting factor is that the participant is a regular user of cannabis and thus an effect of the drug may be present throughout the study. However, he did not report any gap in his usage, allowing us to make the comparison between time points. This will be considered more in detail when comparing results across subjects.

## 5. Conclusions

In conclusion, this case study provides evidence that the efficacy of locomotor training can be significantly enhanced by the addition of scTS as has been shown with epidural implantation [14]. The present observations suggest that the increased effectiveness is attributable to greater recruitment of more motor units during the bursting phase of the muscles and the improved timing of these burst which results in improved coordination to the motor pools and thus muscles. This more normal pattern of activation generates a more normal pattern of proprioception which can, in turn, play a primary role in the spinal networks acquiring a more normal pattern to drive the appropriate motor pools. The degree to which the lumbosacral networks can regain greater control from supraspinal networks after “complete” paralysis remains to be determined. The present data are the result of the first phase of planned studies to further test the extent of recovery that is possible using activity-dependent mechanisms facilitated with noninvasive neuromodulation to recover independent over-ground stepping.

## Data Availability

The de-identified datasets generated through this study can be provided by the corresponding author upon reasonable request.

## Supplementary Materials

The following supporting information can be downloaded at: https://www.mdpi.com/article/10.3390/jcm11133670/s1, Figure S1: Kinematic data collected in a non-disabled volunteer during stepping in a non-weight bearing setting; Figure S2: Effect of rhythmic and tonic scTS on right muscle activity and right knee angle during a voluntary knee extension task; Figure S3: Determination of muscle coordination using peak EMG activity within muscle pairs. Figure S4: Detailed change in interlimb and intralimb coordination from non-weight bearing training to weight-bearing training; Figure S5: Protocol used to evaluate modulation of motor evoked potentials through voluntary tasks; Figure S6: Voluntary modulation of motor evoked potentials during knee and ankle voluntary tasks before and after treadmill training.
