# Supplementary material for: Novel Noninvasive Spinal Neuromodulation Strategy Facilitates Recovery of Stepping after Motor Complete Paraplegia
Source: J Clin Med. 2022 Jun 25;11(13):3670. doi: 10.3390/jcm11133670 (PMC9267673; doi:10.3390/jcm11133670)
Supplement: Supplementary file 1 [file jcm-11-03670-s001.zip › jcm-1692296-supplementary.pdf]

Supplementary Figures - Novel noninvasive spinal neuromodulation strategy facilitates recovery of stepping after motor complete paraplegia

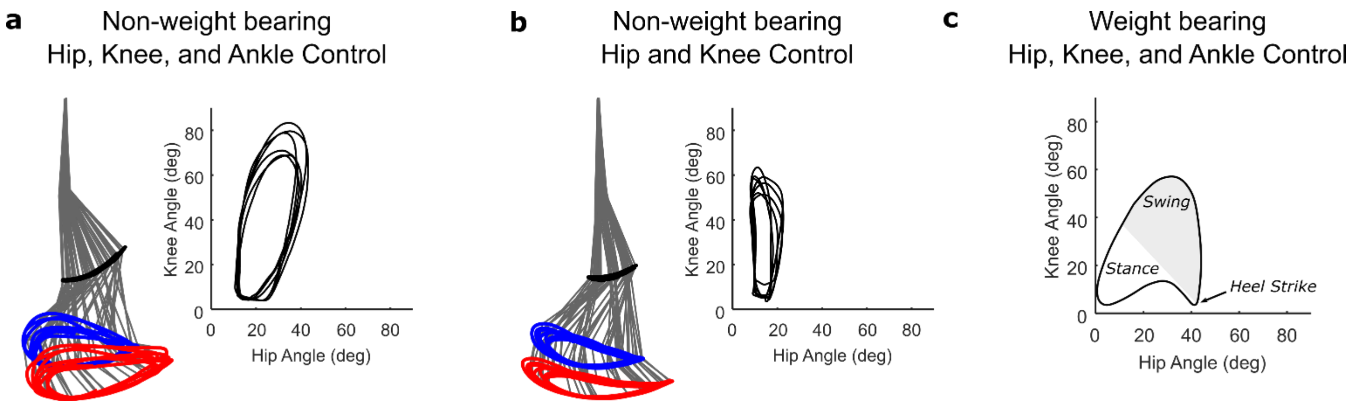

Figure S1. Kinematic data collected in a non-disabled volunteer during stepping in a non-weight bearing setting (**a, b**) compared to a representative cyclogram of weight-bearing stepping (**c**, adapted from Park et. al. 2022). Due to the nature of non-weight bearing stepping, cyclogram features such as heel-strike are missing from the non-weight bearing cyclogram. Likewise, a reduction in hip angle is observed, possibly due to missing proprioceptive cues that signal the initiation of the swing phase and cause an earlier initiation of leg swing.

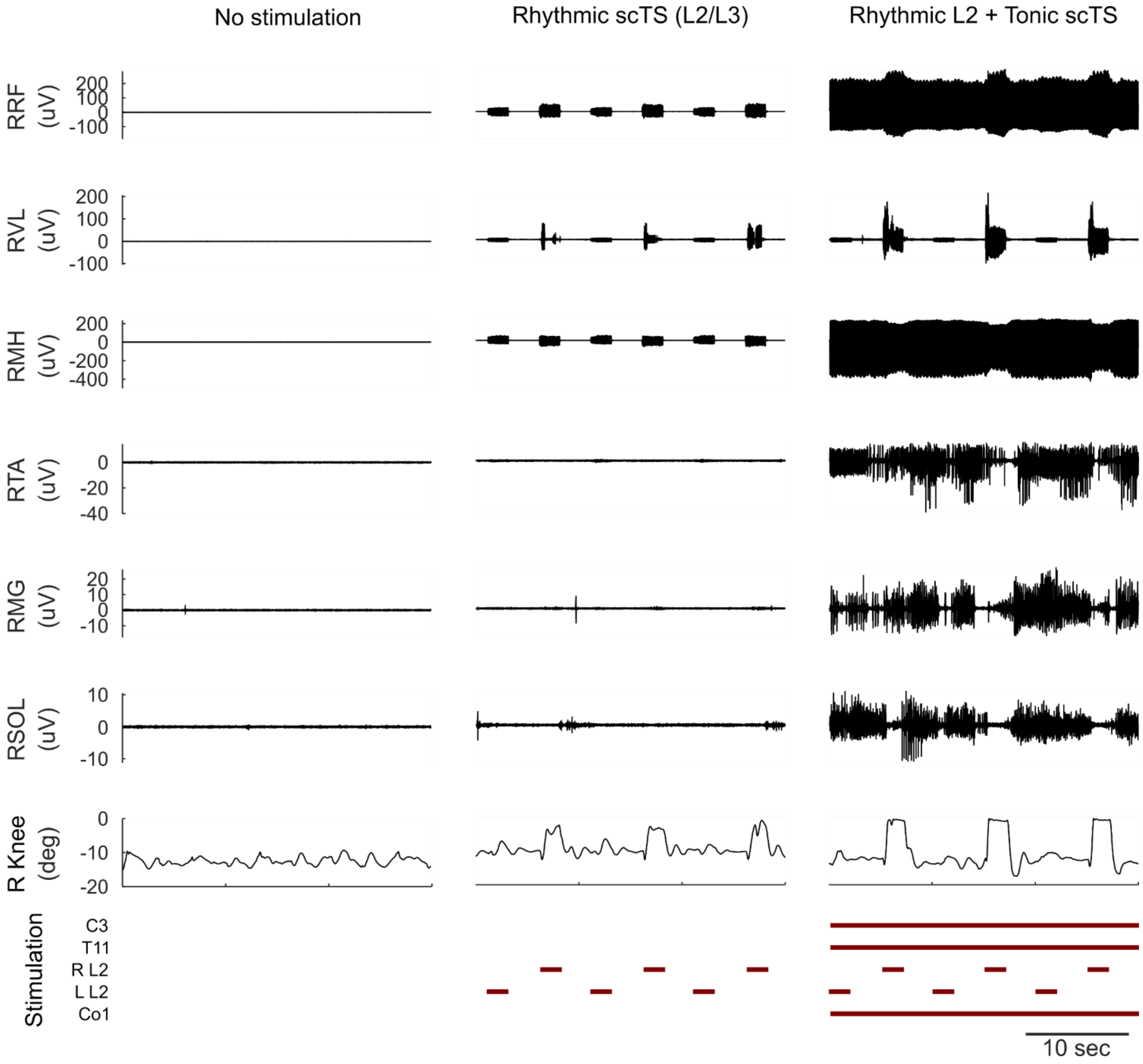

Figure S2. Effect of rhythmic and tonic scTS on right muscle activity and right knee angle during a voluntary knee extension task. Without stimulation, there is no observable voluntary knee extension. However, during scTS there is an increase in the knee extensors (VL and RF), with a significantly greater effect on muscle activity and more stable knee extension when rhythmic scTS is paired with multi-site tonic scTS.

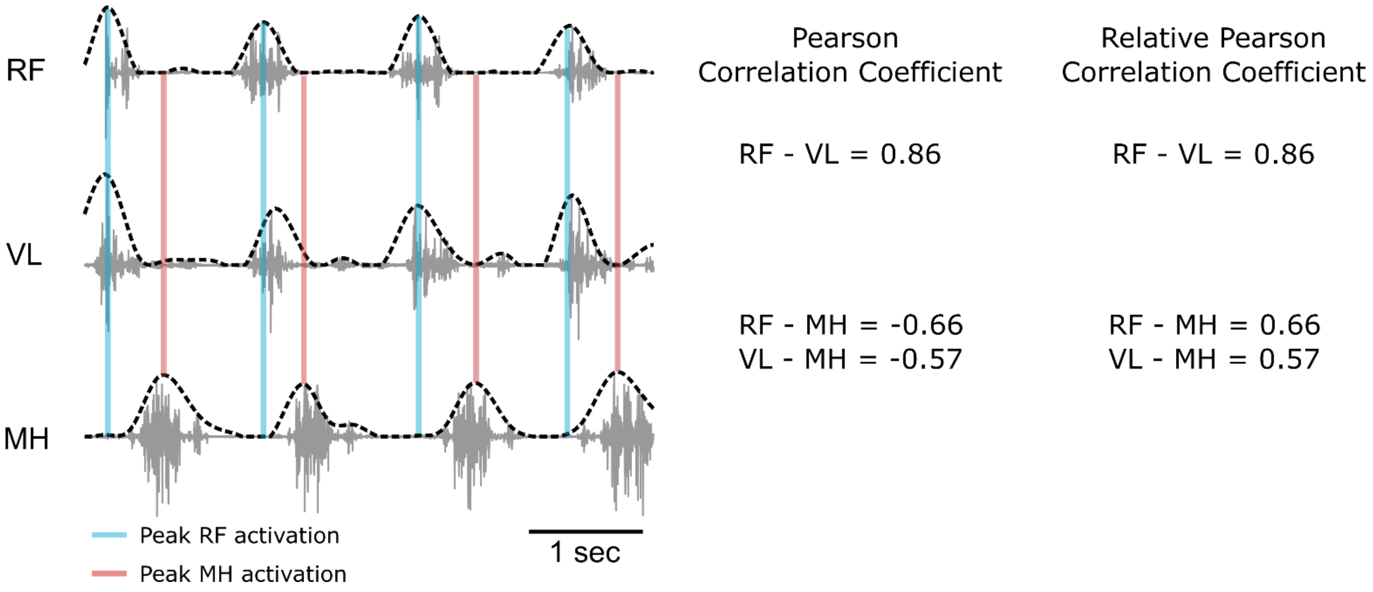

Figure S3. Determination of muscle coordination using peak EMG acitivity within muscle pairs. The Pearson Correlation Coefficients (PCC) between agonist and antagonist muscles were calculated using normalized smoothed EMG values during maximal activation. A relative PCC was obtained by inverting the PCC of all antagonist pairs such that a high positive value indicated adequate coordination and a high negative value indicated inadequate coordination.

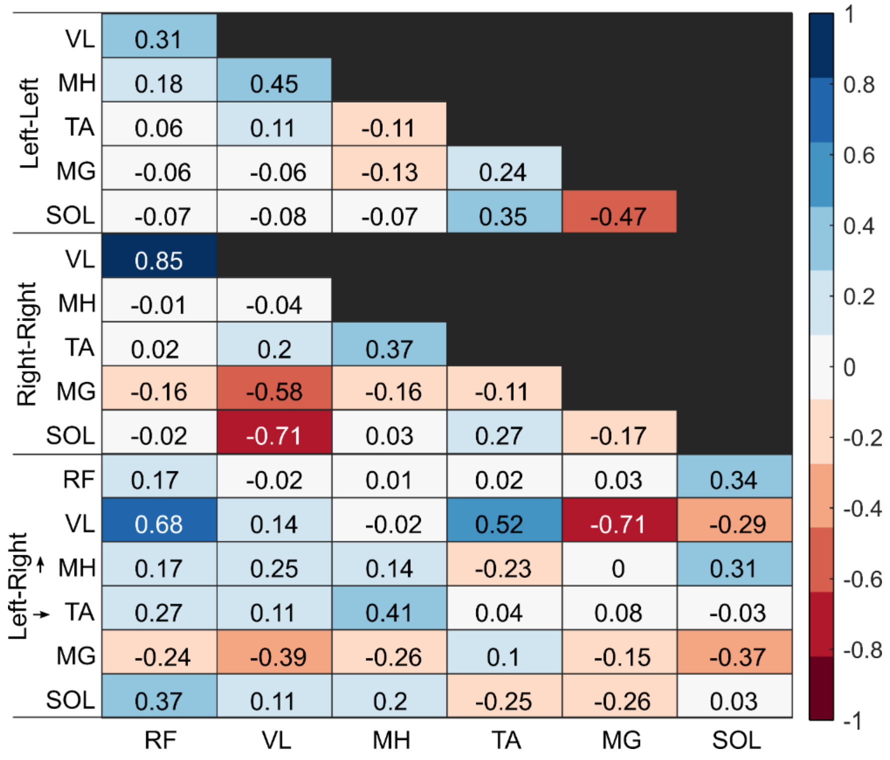

Figure S4. Chart showing the change in interlimb and intralimb coordination from non-weight bearing training to weight-bearing training. The values indicate the difference in relative Pearson correlation coefficient across all muscle pairs from before WBT to after WBT. An increase denotes an increase in coordination between either agonist or antagonist pairs. Inversely, a decrease denotes decreased coordination.

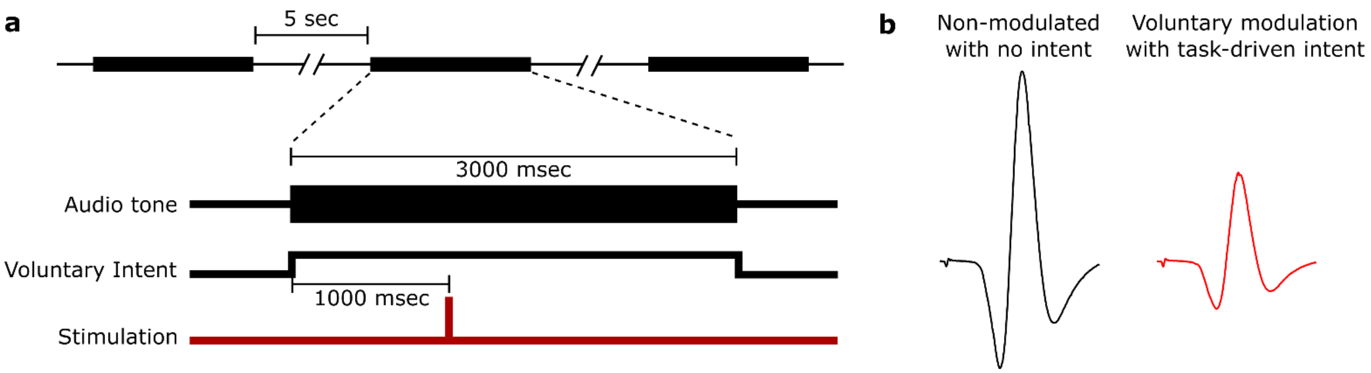

Figure S5. A voluntary motor task is used to evaluate modulation of motor evoked potentials through corticospinal pathways after spinal cord injury. (**a**) The assessment protocol involves the use of an auditory tone to signal the participant to start and maintain a motor task until cessation of tone. A stimulation pulse is delivered to the lumbar spinal cord at L1/L2 1000 msec after the tone to evoke motor responses in all leg muscles. (**b**) Modulation of the motor evoked potential of the vastus lateralis muscle suggests presence of cross-lesional pathways influenced by cortical commands.

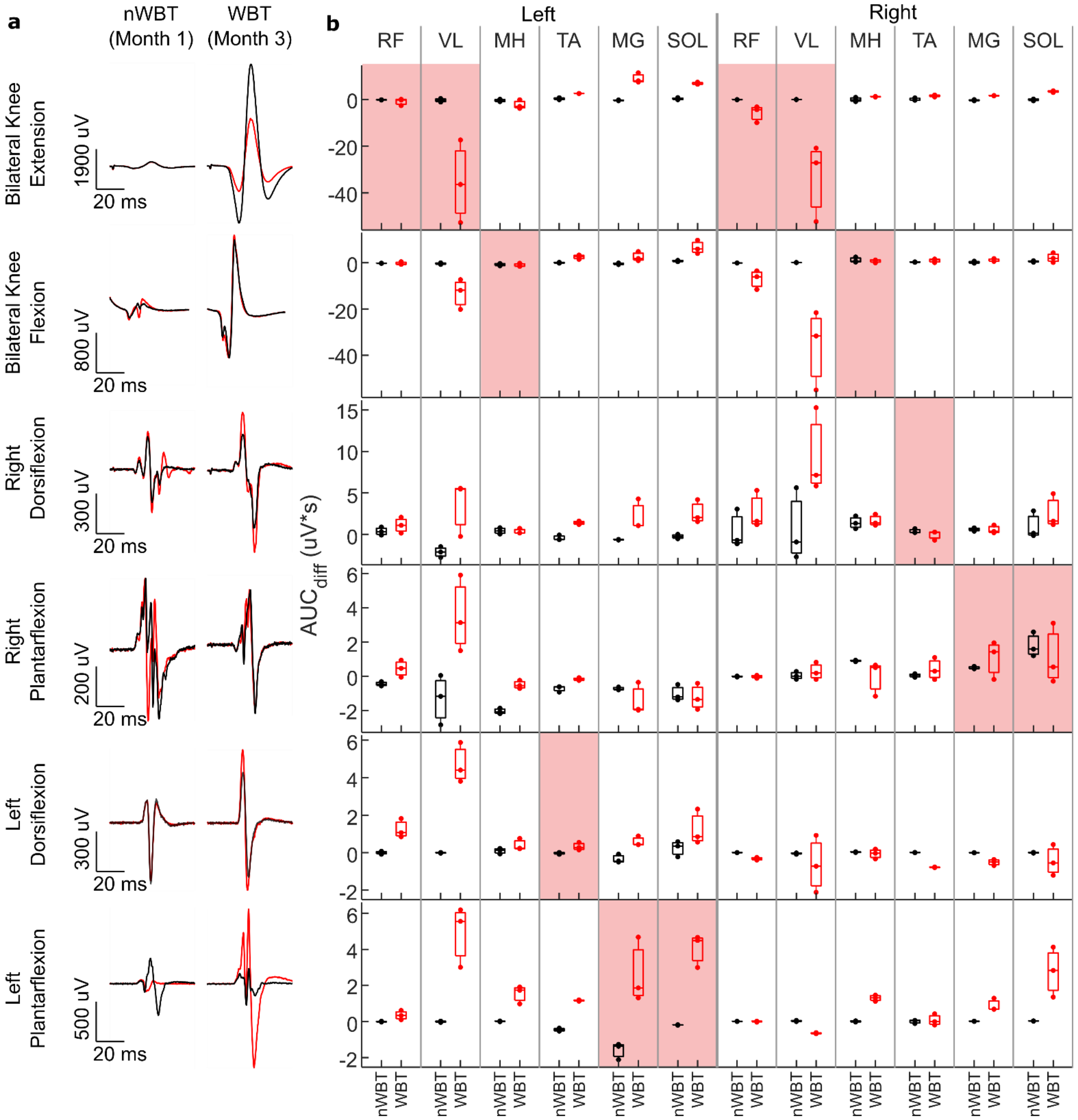

Figure S6. Voluntary modulation of motor evoked potentials during voluntary tasks before and after treadmill training for bilateral knee extension and flexion, and unilateral dorsiflexion and plantarflexion for both legs. (**a**) Spinal cord evoked motor potentials of the main muscle involved in each voluntary task. (**b**) Difference of the area under the curve compared to control pulses across all muscles for each voluntary task. The shaded area indicates the main muscles used for each motor task.
